# Immunotherapy for hypertensive end-organ damage: a new therapeutic strategy

**DOI:** 10.1042/EBC20243000

**Published:** 2025-03-25

**Authors:** Zhiyang Xu, Haisheng Yu, Rulin Zhuang, Qin Fan

**Affiliations:** 1 State Key Laboratory of Flexible Electronics (LoFE) & Institute of Advanced Materials (IAM), Nanjing University of Posts & Telecommunications, Nanjing 210000, China; 2 Department of Geriatrics, The First Affiliated Hospital with Nanjing Medical University, Nanjing 210029, China; 3 Department of Thoracic Surgery, Nanjing Drum Tower Hospital, The Affiliated Hospital of Nanjing University Medical School, Nanjing 210008, China

**Keywords:** end-organ damage, hypertension, immune system, immunomodulation, immunotherapy

## Abstract

Hypertension represents a highly prevalent chronic condition and stands among the foremost contributors to premature mortality on a global scale. Its etiopathogenesis is intricate and multifaceted, being shaped by a diverse array of elements such as age, genetic predisposition, and activation of the neuroendocrine apparatus. Mounting evidence has shed light on the significant part that autoimmune responses play in hypertension and the ensuing damage to end organs. Virtually all varieties of immune cells, spanning both innate and adaptive immune compartments, exhibit a close correlation with the progression of hypertension. These immune cells infiltrate the kidney and vascular mesenchyme, subsequently discharging potent cytokines, reactive oxygen species, and metalloproteinases. This cascade of events can affect the functionality of local blood vessels and potentially precipitate adverse structural and functional alterations in crucial organs like the heart and kidney. In recent times, the management of end-organ damage in hypertension has emerged as a pivotal scientific focus. A multitude of researchers are actively engaged in probing efficacious intervention regimens, among which immunotherapy strategies hold considerable promise and anticipation as a prospective avenue.

## Introduction

**Figure 1:**
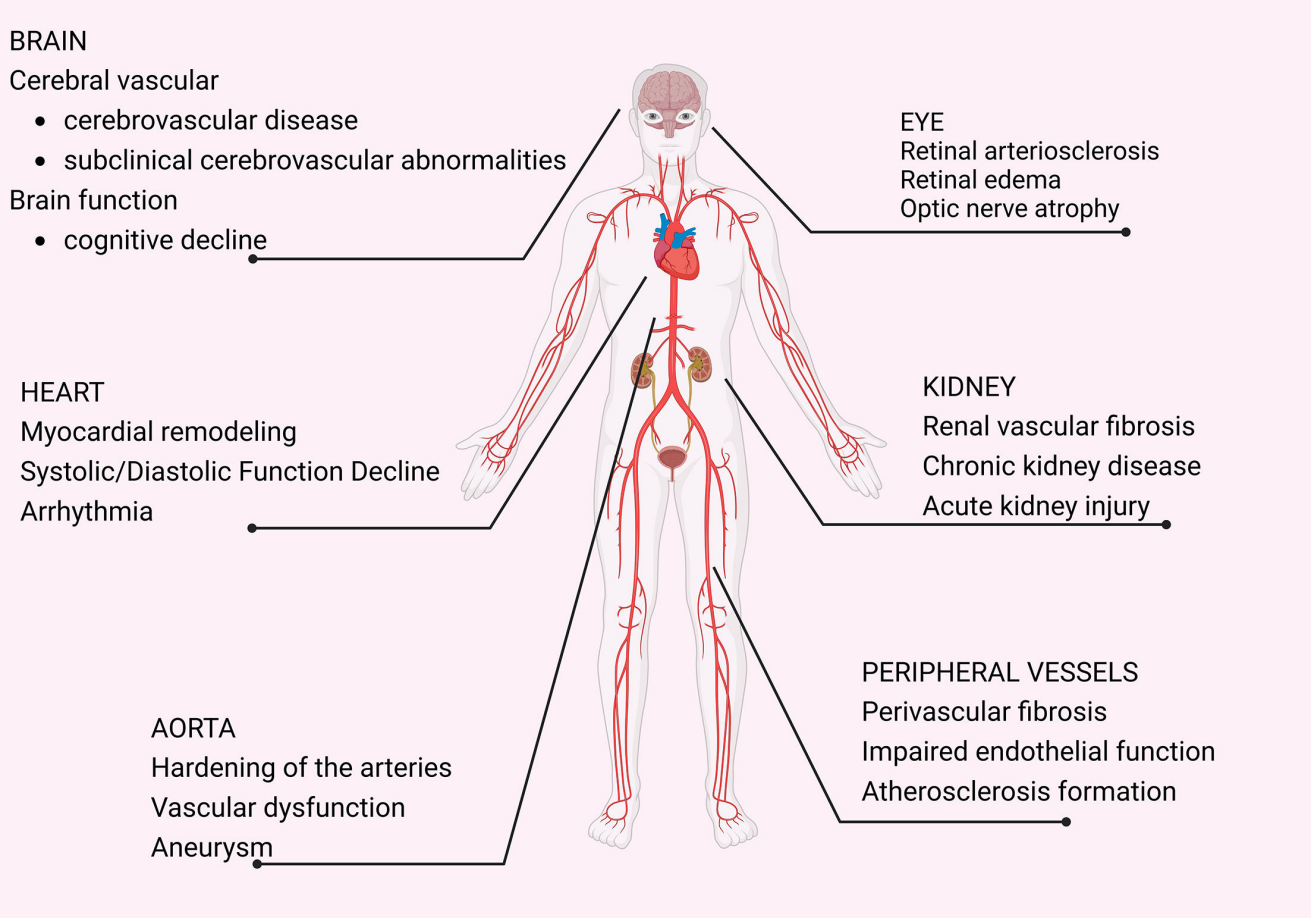
Hypertensive end-organ damage. Hypertension is not only a major risk factor for cardiovascular disease but also leads to a wide range of end-organ damage, including a wide range of organs such as the brain, heart, eyes, kidneys, aorta, and peripheral vasculature, which can lead to conditions such as cerebrovascular disease, cardiac remodeling, retinal problems, renal fibrosis, and vascular dysfunction, which affect the overall health of the patient.

Hypertension is a widespread health concern characterized by a progressive cardiovascular syndrome with multifactorial origins [1–3]. Early signs typically include persistent blood pressure elevation, which heightens the risk of damage to essential organs, particularly the heart, kidney, and vascular system [4–6] (Figure 1). It often induces severe and long-lasting damage to target organs through mechanical stress, inflammatory infiltration, oxidative stress, metabolic disruption, and so on.

Hypertension poses a significant global health challenge, affecting over 1 billion individuals and closely linked to cardiovascular diseases and increased mortality rates. The World Health Organization reports that only 54% of those with hypertension are diagnosed, 42% receive treatment, and a mere 21% achieve effective blood pressure control [7]. This emphasizes the urgent need for improved awareness and management strategies. Recent research highlights the immune system’s crucial role in hypertension and its associated organ damage, paving the way for novel therapeutic approaches [8,9]. Traditionally, hypertension management is centered on pharmacological interventions to protect vital organs by controlling blood pressure [10–12]. However, as our understanding of hypertension’s pathophysiology deepens, particularly regarding the immune system’s involvement, immunotherapy is emerging as a promising strategy to address hypertension-associated organ damage due to its ability to skip blood pressure control and achieve end-organ protective effects [13–16].

In this review, we will explore the potential application of immunotherapy in hypertension-related organ damage, including its mechanism, clinical research progress, and future research directions, with the aim of providing more effective treatment options for hypertensive patients.

### Leveraging immune cells for the treatment of hypertensive end-organ damage

#### Lymphocyte

##### T cells

An imbalance between regulatory T cells (Tregs) and effector T lymphocytes has been implicated in the progression of hypertension and associated vascular injury [17,18]. Enhancing Treg proliferation and function or inhibiting effector T lymphocyte activity through targeted cytokines or antibodies represents a promising therapeutic strategy [19,20]. Studies have demonstrated that low doses of interleukin 2 (IL-2) can selectively expand Tregs, offering protection to organs [21,22]. The AMPK activator metformin is thought to inhibit T-cell proliferation and suppress helper T cells’ (Th1 and Th17) cell differentiation via the AMPK-mTOR pathway or the TIGAR glycolytic pathway, while simultaneously promoting the development of Tregs [23,24]. Its combination with the immunosuppressant tacrolimus might have increased the proportion of Tregs in peripheral blood by nearly 10% and reduced IL-17 levels by approximately 60% in *in vitro* experiments [25]. Preliminary findings suggest that tumor necrosis factor-α (TNF-α) is a significant target for preventing organ damage. The TNF-α antagonist etanercept has been shown to prevent angiotensin II (Ang II)-induced hypertension and the increase in vascular superoxide, with related factors potentially modulating Treg differentiation and function [26,27]. Recent studies indicate that targeting the γδ receptor on T cells may yield more effective results than focusing on the αβ receptor [28]. Additionally, a growing number of studies report that treatments involving estrogen, statins, and vitamin D inadvertently modulate Treg activity [29–31]. These approaches could pave the way for innovative interventions in the management of hypertension-related organ damage.

##### B cells

In hypertensive states, increased B-cell activity may promote an inflammatory response [32,33]. Currently, the main focus for B-cell modulation is on B-cell depletion therapies and B-cell-activating factor inhibition therapies [34]. Monoclonal antibodies against CD20, such as rituximab and orolizumab, have been used to deplete B cells in the treatment of autoimmune diseases. However, their application in the context of hypertensive target organ damage has not been extensively studied due to associated issues, such as humoral immunodeficiency [33,35–37]. Fortunately, research has identified that regulatory B cells can suppress excessive immune responses, thereby promoting tissue repair and attenuating fibrosis through the differentiation of Tregs and the secretion of IL-10 [38,39].

### Macrophages

Macrophages can polarize into different subtypes depending on the microenvironment, mainly comprising the classically activated M1 type and the alternatively activated M2 type [40,41]. M1 macrophages play a promotional role in the inflammatory response, whereas the M2 type is associated with anti-inflammation and tissue repair [42]. Therefore, a promising therapeutic strategy is to use specific cytokines or small molecule drugs to alter the macrophage microenvironment and induce polarization toward M2 [43]. Recent studies have found that AVE0991 (AVE), a Mas receptor agonist, promotes macrophage polarization and inhibits pro-inflammatory immune responses, thereby attenuating salt-sensitive hypertension (SSHTN)-induced blood pressure elevation, inflammation, and end-organ damage [42]. In addition, several conventional hypoglycemic agents, including sodium-glucose cotransporter protein 2 inhibitors, metformin, glucagon-like peptide-1 receptor agonists, and so on, have been shown to potentially affect macrophage polarization, but further studies are needed for the amelioration of end-organ damage due to hypertension [44–47].

### Dendritic cells

Dendritic cells (DCs) are not only involved in the initiation and regulation of immune responses but also play a key role in the pathology of hypertension [48,49]. DCs are important antigen-presenting cells that capture, process, and present antigens, thereby activating T and B cells and initiating an adaptive immune response [50,51]. Excess sodium ions enter DCs in a state of hypertension induced by a high-salt diet, which may promote their maturation and activation and, thus, exacerbate the inflammatory response associated with hypertension [52]. DCs not only produce pro-inflammatory cytokines, such as monocyte chemoattractant protein 1, TNF-α and IL-6, but also drive the secretion of pro-inflammatory cytokines by activated T cells, which affect vascular tone and endothelial function, ultimately leading to target organ damage [53,54]. The maturation and activation of DCs can be inhibited by inhibiting the NF-kB, PI3K, and MAPK pathways, thus reducing their pro-inflammatory effects [55,56]. Unlike other immune cells, sodium intake may be controlled to reduce its effects on DCs, thereby reducing the incidence of hypertension and the risk of target organ damage [48].

### Natural killer cells

Natural killer (NK) cells are part of the innate immune system and can cause structural and functional damage to target organs by promoting local inflammation and apoptosis [57]. Studies have shown that Ang II induces NK cell recruitment in the aortic wall, and this recruitment may exacerbate inflammatory responses and damage to the vasculature [58–60]. Several studies have indicated that NK cells can influence cardiac remodeling and function by regulating the interaction of the chemokine CCL5 with T cells, while producing macrophage colony-sensing factor (m-CSF) and macrophage inflammatory protein 1α and -β (CCL3 and CCL4), which recruit and activate macrophages and further contribute to the inflammatory response [61–63]. Although NK cells may play a role in promoting inflammation and injury in hypertension, studies have also explored the potential protective role of NK cells. CD1d-dependent NKT cells showed protective effects in hypertension and cardiac remodeling, suggesting that different types of NK cells may play different roles in hypertension [64].

### Therapeutic strategies based on immune molecular regulation

**Figure 2:**
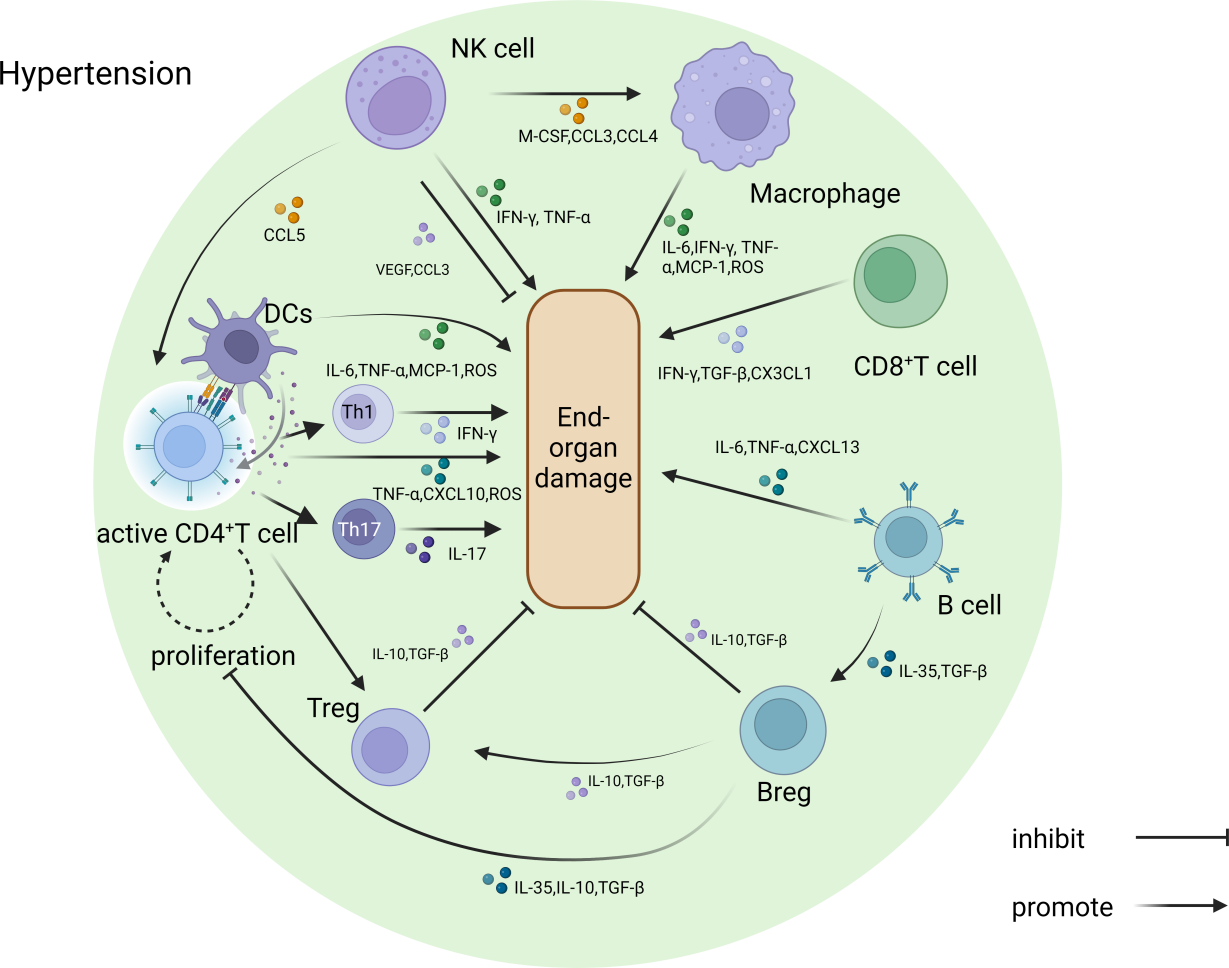
Role of various immune cells and immune molecules in hypertensive end-organ damage. In the pathological environment of hypertension, CD4+ T cells and their subpopulations, CD8+ T cells, B cells, macrophages, dendritic cells, and NK cells interact with each other through various factors such as IFN-γ, TNF-α, and cytokines such as IL-6, thus affecting target organs. IFN, interferon; NK, natural killer; VEGF, vascular endothelial growth factor.

Although immune cells are the mainstay of the immune responses, more often than not, the immune molecules they secrete and release have the direct effects. Cytokines are the predominant signaling molecules secreted by immune cells. In response to infection and inflammation, the immune system releases a variety of cytokines that play pivotal role in the development of hypertension [65–68]. Studies have shown a direct correlation between immune cells and the cytokines they secrete and elevated blood pressure and organ damage [60,66]. TNF-α, interferon-γ (IFN-γ), IL-6, and IL-17 are the key pro-inflammatory cytokines, which contribute to hypertension by promoting inflammation and triggering vasoconstriction [69,70]. Cytokine inhibitors may attenuate hypertension-related organ damage by reducing the activity of specific cytokines. Many cytokine inhibitors are based on monoclonal antibodies that specifically target and neutralize pro-inflammatory cytokines. The TNF-α blocker etanercept and the IL-1 receptor anakinra have shown antihypertensive and renal antifibrotic effects [71,72]. Recombinant IL-10 may protect end organs by reducing proliferation of activated T cells and endothelial cell inflammation [73]. In addition, monoclonal antibodies against IL-6 and IL-17 have shown potential benefit in hypertensive patients in several studies [18,74] (Figure 2).

Chemokines represent a specialized class of cytokines that play a significant role in the pathology of hypertension, particularly in relation to hypertension-related organ damage [65,75,76]. m-CSF is a chemokine that regulates the recruitment and activation of monocytes and macrophages. Research has shown that oxidative stress and inflammatory responses induced by Ang II are significantly attenuated in m-CSF-deficient mice [77]. CCR2b inhibitor protects kidneys and slows the progression of hypertension by reducing inflammatory response, suggested by the Elmarakby et al. study [78]. In addition, the chemokine CX3CR1 has been found to have a protective role in hypertension-induced renal injury by modulating the invasion of inflammatory cells [79]. These findings indicate that targeting specific chemokine receptors may offer new strategies for treating hypertension-related organ damage. While the clinical application of chemokine inhibitors is still in its early stages, their potential therapeutic value should not be overlooked.

### Anti-inflammatory drug-based treatment strategies

Recent studies underscore the critical role of inflammatory mechanisms in blood pressure regulation, engaging a range of immune cells [80–82]. Inflammatory cells and mediators cause vasoconstriction, impaired diastole, and vascular remodeling by releasing pro-inflammatory cytokines and triggering immune responses [59,65]. The incorporation of anti-inflammatory drugs into hypertension management is gaining traction as a promising therapeutic strategy. The classical antihypertensive drugs Ang-converting enzyme inhibitors and Ang II receptor blockers have been found to not only lower blood pressure by inhibiting Ang-converting enzyme and reducing Ang II production but also exert an anti-inflammatory effect by inhibiting inflammatory factors and reducing oxidative stress, which in turn protects end organs [83–85]. Statins and vitamins reduce hypertension-induced vascular inflammation and organ damage by inhibiting inflammatory signaling pathways and stabilizing endothelial function [86,87]. Notably, the multiple anti-inflammatory properties of some non-steroidal anti-inflammatory drugs, which inhibit not only cyclo-oxygenase activity to suppress inflammation but also the release of leukotrienes and various pro-inflammatory cytokines, may play a key role in end-organ protection in hypertension [88]. Additionally, novel anti-inflammatory agents, such as developmental endothelial locus 1, have demonstrated the ability to lower blood pressure and protect target organs by inhibiting inflammatory responses and enhancing vascular function [26].

### Therapeutic strategies based on vaccines and stem cell therapies

#### Hypertension vaccine

Hypertension vaccines that are currently under development mainly target the renin-Ang system (RAS) component and have been shown to have significant protective effects against hypertension-induced arterial and renal damage. Ang I-R vaccine against the endogenous peptide Ang I significantly reduced systolic blood pressure (−15 mmHg) after a single injection in a rat model of spontaneous hypertension [89]. The pHAV-4Ang IIs vaccine and the CYT006-AngQb vaccine reduce blood pressure and improve organ function by inducing specific antibodies against Ang II and blocking the binding of Ang II to its receptor [90–92]. In contrast, the ATR12181 vaccine and ATRQβ-001, which target antibodies against the Ang II type 1 receptor, are considered more promising for lowering blood pressure and reducing left ventricular hypertrophy and fibrosis [93–95]. Therapeutic vaccination against RAS components is expected to overcome the need for daily medication and provide long-term patient compliance-enhancing effects.

#### Stem cell therapy

Stem cells are a class of cells with self-renewal ability and multidirectional differentiation potential and are capable of differentiating into many types of cells under specific conditions. Due to their excellent regenerative and anti-inflammatory abilities, stem cells have attracted much attention in the field of cardiac regeneration [96,97]. Stem cells can differentiate into cardiomyocytes and other cell types to improve heart function, reduce myocardial fibrosis, and protect the kidneys, thereby reducing organ damage caused by high blood pressure [98]. However, stem cells have not been used in the prevention and treatment of hypertensive target organ damage due to theoretical problems and immune rejection issues.

#### Future outlook

The major difficulties in immunomodulatory therapy for end-organ damage in hypertension lie in two aspects: first, how to control the ‘degree’ of immunomodulation and secondly how to achieve precise targeting of immunotherapy. Above all, comprehensive assessment before treatment, rational use of medication during treatment, and close monitoring after treatment will be important means to avoid secondary damage caused by immunotherapy, and the use of these means is more dependent on the subjective judgment of the healthcare provider. How to carry out organ targeting without involving other organs is more inclined to objective development [99,100]. A recent *ACS Nano* article on ‘Particle-Based Artificial Antigen-Presenting Cell Systems’ offers new ideas for end-organ protection in hypertension. The system precisely stimulates the activation of specific T cells by artificial antigen-presenting cells based on biomaterials, in particular particles and nanoparticles [101]. This suggests that combining nano-engineering with immune cell-specific targeting strategies may break through the current efficacy bottleneck of end-organ injury treatment [102,103].

## Conclusion

Hypertension-induced organ damage represents a significant public health challenge, affecting critical organs such as the heart, brain, and kidney. While existing studies have highlighted the severe consequences of hypertension, numerous research avenues and challenges remain underexplored. Overcoming the methodological, resource-related, and clinical translation obstacles is essential for advancing our understanding of this complex issue. Through dedicated research and interdisciplinary collaboration, we anticipate making substantial strides toward mitigating hypertension-related organ damage in the future.

**Summary**

- Hypertension is a widespread health concern that damages vital organs, including the heart and kidney, while leading to severe complications.
- The immune system significantly contributes to hypertension-related organ damage, unveiling new therapeutic avenues beyond traditional pharmacological approaches.
- Ongoing research examines various immune cells—such as T cells and macrophages—and their roles in hypertension, paving the way for innovative immunomodulatory treatments targeting cytokines and chemokines.
- While immunotherapy holds promise, challenges remain, particularly the complexity of hypertension's multifactorial nature and the need to bridge the gap between basic research and clinical applications.

## Abbreviations

DCs: dendritic cells
IFN: interferon
IL: interleukin
TNF-α: tumor necrosis factor-α
Tregs: regulatory T cells
m-CSF: macrophage colony-stimulating factor
